# Opportunities for Consistent and Holistic Metrics to Support Food Systems Transformation: A Summary of a Symposium Presented at Nutrition 2023^☆^

**DOI:** 10.1016/j.cdnut.2024.102129

**Published:** 2024-03-05

**Authors:** Allison L Unger, Ty Beal, Zach Conrad, Matthew A Pikosky, Katie Brown

**Affiliations:** 1 National Dairy Council, Rosemont, IL, United States; 2 Global Alliance for Improved Nutrition, Washington, DC, United States; 3 Department of Kinesiology, William & Mary, Williamsburg, VA, United States; 4 Global Research Institute, William & Mary, Williamsburg, VA, United States

**Keywords:** Access to Nutrition Index, agrobiodiversity, bioavailability, Global Nutrition Report Nutrition Accountability Framework, micronutrients, National Strategy on Hunger, Nutrition, and Health, NHANES, food-based dietary guidance, regenerative agriculture, World Benchmarking Alliance

## Abstract

There is an urgent need for global food systems transformation to realize a future where planetary health reaches its full potential. Paramount to this vision is the ability of stakeholders across sectors to understand how foods and dietary patterns impact food systems inclusive of all domains of sustainability—environmental, nutrition/health, economic and social. This article is a synopsis of presentations by 3 food systems experts to share the latest science in a session entitled “How do you measure sustainability? Opportunities for consistent and holistic metrics to support food systems transformation” at the American Society for Nutrition’s 2023 annual conference. As summarized here, global population data showing widespread malnutrition underscore the important role of dietary diversity through a balance of plant- and animal-source foods to achieve nutritionally adequate diets and reduce risk of noncommunicable diseases. Yet, recent international audits of countries, companies, and organizations and their sustainability targets largely demonstrate an underrepresentation of robust nutrition/health metrics to support public nutrition and health progress. Addressing limitations in diet-sustainability modeling systems provides a viable opportunity to accurately reflect the important contributions and trade-offs of diets across all domains of sustainability to ultimately support evidence-based decision making in advancing healthy food systems.

## Introduction

Now more than ever, there is an urgent need to develop and employ strategies to achieve planetary health (i.e., “the health of human civilisation and the state of the natural systems on which it depends” [1]). Such food system transformation will require stakeholders across sectors to holistically grapple with the synergies and trade-offs that will necessarily occur when making decisions regarding food production and sustainability targets. Yet, requisite to such discussions is the availability of a robust body of literature assessing the 4 domains of sustainability—environmental, nutrition/health, economic, and social—and respective measurement tools and models that allow for the integration of 2 or more domains to quantify those synergies and trade-offs. This arena of research is notably still in its infancy, and more work is critically needed.

This article is a synopsis of a symposium entitled “How do you measure sustainability? Opportunities for consistent and holistic metrics to support food systems transformation” presented at the American Society for Nutrition’s 2023 annual conference. This symposium session featured presentations by 3 food systems experts who shared the latest science to discuss the complementary nature of plant- (PSF) and animal-source food (ASF) to population health, with a focus on the role of nutrition/health frameworks to accurately capture these important nutrition and health contributions. Additionally highlighted within this symposium were key considerations and opportunities for designing and evaluating robust diet-sustainability modeling to better account for the synergies and trade-offs of future sustainable diets.

## Understanding the Role of Dietary Diversity in Sustainable Food Systems

### The global burden of malnutrition

Too many people in the world are malnourished [2]. Estimates of prevalence of undernutrition and diet-related noncommunicable diseases emphasize the stark reality that, worldwide and across countries of all income levels, people are not getting enough of the nutrients they need and are frequently consuming excess Calories [2]. According to the 2021 Global Nutrition Report (GNR), which provides independent assessment of indicators on global nutrition [2], 22% of all children are stunted and 6.7% are wasted, with 14.6% of live births being low birth weights. At the same time, these data show that 5.7% of children globally are overweight. Among adults, 9.1% and 8.1% of females and males, respectively, are underweight, whereas diet-related cardiometabolic risk factors and disease are widely prevalent. Notably, 40.8% of females and 40.4% of males are overweight; 19.9% of females and 24.0% of males have elevated blood pressure; and 8.9% of females and 10.5% of males have diabetes, respectively [2].

Recent global estimates that directly evaluate measures of diet quality reinforce that micronutrient deficiencies are a significant public health concern. In 2022, Stevens et al. [3] reanalyzed individual-level biomarker data for micronutrient status from nationally representative, population-based surveys to estimate the global and regional prevalence of deficiency in ≥1 of 3 micronutrients among preschool-aged children (aged 6–59 mo) and nonpregnant females of reproductive age (aged 15–49 y). These analyses found that 56% of preschool-aged children (372 million) and 69% of females of reproductive age (1.2 billion) have 1 or more micronutrient deficiencies. Examination of the data demonstrated region-specific, yet nevertheless pervasive, prevalence of these micronutrient deficiencies globally, including both low- and high-income countries. For example, 9 in 10 females in countries in South Asia and Sub-Saharan Africa, 1 in 2 females in the United Kingdom, and 1 in 3 females in the United States were reported to have ≥1 micronutrient deficiency. Of note, iron deficiency was estimated to affect 1 in 5 females in the United Kingdom and United States [3]. Other work published in the same year supports these findings, highlighting that the prevalence of dietary micronutrient inadequacy is ubiquitous worldwide, and often that a regional population is affected by overlapping inadequacies in several nutrients critical for optimal health [4]. Ongoing research suggests that micronutrient inadequacies are likely different between males and females with certain nutrients showing greater inadequacy among males and others among females [4], attributed to sex-specific differences in both nutrient requirements [5] and dietary habits [4]. Beyond vitamins and minerals, evidence of insufficient macronutrient consumption is evident as well, with >1 billion people not meeting recommended protein intakes globally [6]. Overall, it is clear that the current food system is inadequate to meet the needs of our current—and growing—global population.

### Achieving adequate nutrition and health through a balance of PSFs and ASFs

Although poor diet quality is driving nutrient deficiencies on a global scale, specific nutrient inadequacies and their principal underlying causes differ across high- compared with low-income countries. Consumption of nutrient poor, energy-rich foods has been proposed as a contributing factor of malnutrition in high-income countries [7,8]. In the United States, 22% and 14% of females of reproductive age are deficient in iron and zinc, respectively [3], and 89% of Americans aged ≥2 y are below adequate intakes of choline [9]. Inadequate intake of vitamin E, magnesium, and calcium are also found in approximately half or more of females [4].

In contrast, regions including Sub-Saharan Africa and South Asia are characterized by low-dietary diversity because of underlying barriers impairing readily affordable and accessible nutrient-dense foods. Data from global surveys show that eating patterns in these regions include low consumption of fresh fruits and vegetables, such as leafy greens, and higher consumption of starchy staples [10]. Few ASFs are consumed, with estimates at ∼1 serving or less per day of any ASFs, such as milk and meat products or eggs [11].

Low-ASF consumption is concerning, because ASFs are dense sources of many essential nutrients and several specific compounds that are not found in PSFs, including retinol, heme iron, and vitamin B12, as well as vitamin D with the exception of certain mushrooms. A recently developed aggregated global food composition database by Beal and Ortenzi [12] demonstrates that ASFs and dark leafy greens are top sources of commonly lacking micronutrients in global diets (vitamin A, folate, vitamin B12, calcium, iron, and zinc). Furthermore, ASFs provide more bioavailable forms of essential nutrients, such as iron and zinc because of the lack of anti-nutrients present and, for iron, the presence of heme iron [13]. The bioavailability of vitamin A in ASFs compared with PSFs has been reported as a 12:1 difference on average [13]. Animal-source protein is also generally of higher quality than plant-source protein, with the exception of soy protein, because of providing the complete array of essential amino acids in adequate amounts and these amino acids being more digestible/bioavailable [13].

At the same time, current diets are globally insufficient in diverse and nutrient-dense sources of PSFs, such as fresh fruits and vegetables, legumes, nuts, and seeds [10]. PSFs, such as ASFs, provide complementary nutrients to the diet that are essential for proper nutrition and health promotion. Importantly, PSFs are the only sources of dietary fiber, the only significant source of vitamin C and among top sources of folate, magnesium, potassium, and phytonutrients [13,14]. Such an imbalance in the quantity and quality of ASF and PSF consumption globally is both a symptom and an underpinning of the current global food system that is essential to address to make strides toward planetary health.

### The role of dietary diversity for planetary health

Stakeholders across sectors seeking to identify viable solutions to address climate change commonly implicate production of ASFs as the primary underlying cause of observed negative environmental impacts, including greenhouse gas emissions (GHGe), land use, soil health, water quantity and quality, and biodiversity [13]. However, this narrative does not consistently include the unintended negative consequences that may occur by limiting ASF consumption, despite research that provides evidence of the challenges that occur with low-ASF consumption to meet a nutritionally adequate diet. Recent modeling of micronutrient intakes with adherence to the planetary health diet proposed by the EAT-Lancet Commission found that diets of females of reproductive age would be 55% below recommended intakes for iron, mostly because of limitations in the density and bioavailability of iron within PSFs [15]. Accordingly, there is a need within the global dialog to revolutionize and align on the metrics we employ to evaluate the role of diets within the food system across multiple domains of sustainability, inclusive but beyond environment. Existing metrics that quantify the environmental impact (GHGe, land and water use, etc.) of a food are useful but simplistic, and importantly do not holistically capture the nutritional/health value of a food or the link between dietary diversity and planetary health [16].

Agrobiodiversity has received increasing attention as a strategy to support sustainable foods systems through dietary diversity by providing nutrient adequate, healthy diets while enhancing resilience, conserving genetic resources, maintaining ecosystem services, and preserving cultural heritage [17]. Livestock such as ruminants, when managed responsibly, can be a beneficial contributor to agrobiodiversity and environmental health. For example, although pasture and rangeland use is required for ruminant livestock production, much of this land is unsuitable for crop production; furthermore, livestock play an important role in nutrient cycling that can promote biodiversity and habitat maintenance [13,18].

Taken together, global data underscore that current food systems are facing challenges in meeting public nutrition and health needs. Malnutrition affects billions globally, with poor dietary quality the driving commonality. Food systems likely need to sustainably produce a diversity of ASFs and PSFs through implementation of responsible agriculture practices, such as agroecology and circular agroecosystems, increased efficiency, and reduced food loss and waste, to nourish people and the environment. To do so can be facilitated by a fuller understanding of the impact of food production and diets on food systems with holistic metrics that consider the integration of nutrition and health with environmental sustainability as well as economic and social domains.

## An Assessment of Frameworks that Inform Global Commitments to Advance Food System Transformation

### Current landscape for goals and commitments by countries, companies, and organizations

The launch of the UN Sustainable Development Goals (SDGs) in 2015 called for transformation of the global food system by 2030 to achieve a sustainable food supply that is nutritious, environmentally responsible, equitable, and affordable [19]. This was followed by galvanized support of UN member countries and led to a nearly unanimous adoption of the SDGs. The year 2021 marked another critical moment with international recognition to incite progress toward SDG targets when the UN held its first ever Food Systems Summit, coinciding with the 2021 UN Climate Change Conference and the Tokyo Nutrition for Growth Summit. These events cumulatively established the essentiality of food systems transformation and called for commitments across the public and private sectors to demonstrate progress toward achieving the SDGs. Yet, such a rapid rise in these commitments has generally been conducted by regions and countries “in a silo” rather than through integrated and collective approaches, with a resultant lack of consensus of best practices on “what” and “how” to mobilize and measure food systems transformation progress.

The FAO defines sustainable diets as “diets with low-environmental impacts which contribute to food and nutrition security and to healthy life for present and future generations…protective and respectful of biodiversity and ecosystems, culturally acceptable, accessible, economically fair and affordable; nutritionally adequate, safe and healthy; while optimizing natural and human resources” [20]. On the basis of these guiding principles, a leading framework to conceptualize and discuss the key elements of a sustainable food system has emerged characterizing 4 overarching and interdependent domains: environment, nutrition/health, economic, and social [21]. This holistic understanding of the multifaceted nature of food systems has important implications, because decisions based on any one food system domain may result in unintended consequences to other domains.

### Prominent frameworks for nutrition and health targets

**FIGURE 1:**
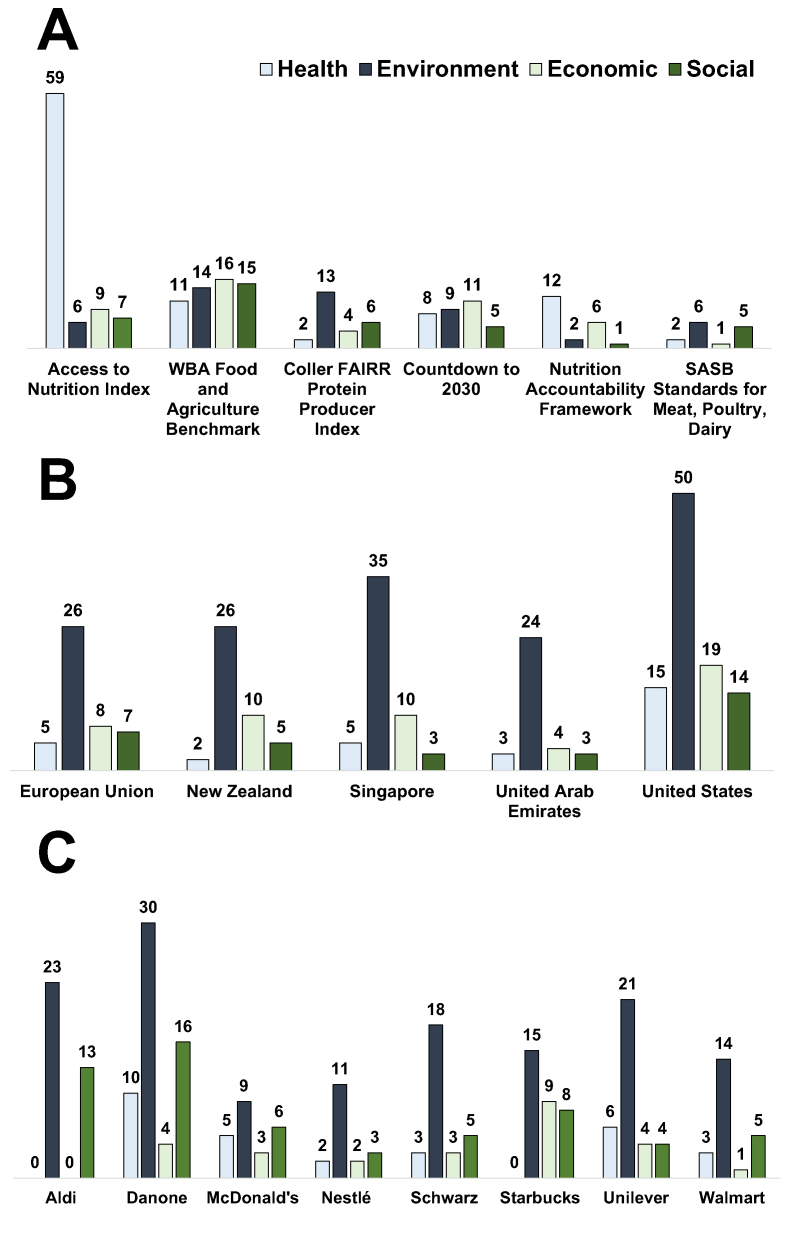
Summary of a landscape audit of prominent frameworks available to assess nutrition and health targets (A) and goals and commitments made by countries (B), companies and organizations (C). Audit results are categorized by an internationally recognized framework defining 4 domains of sustainable food systems [21]. FAIRR, Farm Animal Investment Risk and Return; SASB, Sustainability Accounting Standards Board; WBA, World Benchmarking Alliance. Figure adapted based on previously published research with permission and/or publicly available data: (A) [22]. (B) European Union [[24], [25], [26], [27]]; New Zealand [[28], [29], [30], [31], [32]]; Singapore [33]; United Arab Emirates [[34], [35], [36]]; Unites States [26,37]. (C) [22]; Aldi [38,39]; Danone [[40], [41], [42]]; McDonald’s [43]; Nestle [44]; Schwarz [45]; Starbucks [46]; Unilever [47]; Walmart [48,49].

To better understand the measurement systems in play at a global level, Miller et al. [22] completed a landscape audit of food systems transformation commitments and measurement frameworks that track progress toward those commitments according to the 4 domains of sustainability (i.e., environment, nutrition/health, economic, and social). This audit revealed a staggering amount of frameworks in this space; as of 2021, there are >600 environmental, social, and corporate reporting provisions globally, and this number has approximately doubled within the past 5 y [23]. Thus, this audit selected frameworks relevant to the food and agriculture sector for evaluation that are led by an authoritative/credible third-party source, report results with regular cadence (e.g., annually or biannually), and provide publicly available data (Figure 1A) [22,[24], [25], [26], [27], [28], [29], [30], [31], [32], [33], [34], [35], [36], [37], [38], [39], [40], [41], [42], [43], [44], [45], [46], [47], [48], [49]].

The audit showed that there are several nutrition/health metrics being employed by third-party frameworks relative to the other domains of sustainable food systems. In particular, 3 frameworks that capture the most nutrition/health metrics include Access to Nutrition Index (ATNI), World Benchmarking Alliance’s (WBA) Food and Agriculture Benchmark, and GNR Nutrition Accountability Framework (NAF) (Figure 1A). This audit also demonstrated that overall representations of nutrition/health metrics were somewhat inflated because of the single third-party framework ATNI (Figure 1A). ATNI, which evaluates food and beverage companies aiming to increase accountability on nutrition and health commitments made by the private sector [50], was found to be the most extensive framework with a total of 81 indicators, 59 of which relate to nutrition/health. Thus, the presence of nutrition/health metrics within the audit was dramatically reduced with ATNI removed from the analysis. ATNI was also noted to have very few indicators addressing the environment or social domains, and environmental metrics were more prominently represented on average across other third-party frameworks (Figure 1A). The authors identified WBA Food and Agriculture Benchmark as the most comprehensive framework, with 56 indicators relevant across all 4 domains of sustainability and 11 metrics focusing on nutrition/health [22]. WBA Food and Agriculture Benchmark measures and ranks 350 of the world’s most influential food and agriculture companies on their contributions to transforming the global food system [51]. Of note, WBA Food and Agriculture Benchmark is updated biannually, with the first edition released in 2021 and the second one scheduled to be released this year.

The GNR NAF also has one of the most robust frameworks to capture nutrition commitments from global food systems stakeholders, employing “Specific, Measurable, Achievable, Relevant and Time-Bound” guidelines to track 12 nutrition/health metrics [22,52]. NAF, endorsed by the WHO, UNICEF, U.S. Agency for International Development (USAID), and Scaling Up Nutrition Movement, was primarily developed to serve as an accountability tool for commitments and actions pledged during the 2021 UN Food Systems Summit and Nutrition for Growth Summit. Importantly, the 2022 GNR provides an analysis of commitments made from a wide range of stakeholders, highlights possible gaps, and develops recommendations based on focus areas where greater action is needed [52]. In addition, starting in 2022, the GNR began to track the annual progress of all registered commitments through a self-reporting survey tool; as of June 2023, 433 commitments had been received by 205 stakeholders [52]. Taken together, the NAF’s vision and objectives position this framework as a desirable accountability tool for nutrition/health targets.

### How nutrition and health metrics provide an opportunity to support food system transformation and human health

An updated audit for country-level food systems commitments, based on the parameters defined within Miller et al. [22], was reviewed to better understand how countries and regions are aligning sustainability progress with SDG targets (Figure 1B; unpublished observations from publicly available data). To ensure a well-rounded audit, a mix of countries that were geographically diverse and/or made food systems, nutrition, and/or climate commitments were assessed. The audit demonstrated a disproportionate prioritization on country-specific environmental commitments and less emphasis on nutrition, economic, and social commitments. Country-level nutritional commitments assessed were narrowly focused on nutrition security. For example, the United States has pledged to ensure access for every child to nutritious school meals via the Coalition for School Meals by 2030 [53], whereas the European Union will invest €2.5 billion over 3 years for international cooperation to address malnutrition by 2024 [54]. Comparatively, environmental commitments span diverse topics, such as GHGe, methane emissions, energy, climate-smart agriculture, natural resources, and biodiversity. Social goals from countries focus on improving youth empowerment and engaging indigenous populations. Economic commitments largely center on trade, such as the United Arab Emirates’ goal of having a global network of partners and investments that enable the safe, reliable, and cost effective supply of food and agriculture products necessary to complement domestic production by 2051 [55].

Lastly, several companies (inclusive of a mix of retailers, consumer packaged goods companies, multinational corporations, and quick-service restaurants) were audited for their sustainability goals [22]. Miller et al. [22] observed that private sector companies generally track their own contributions related to sustainability, with most emphasizing environmental commitments; an updated evaluation of these companies for this symposium session confirms that this reporting pattern has not markedly changed (Figure 1C; unpublished observations from publicly available data). The company audit tended to align measurement and reporting structures with 1 or more third-party frameworks. Environmental goals were generally more quantitative whereas nutrition/health, social, and economic commitments tended to be more qualitative in nature and less likely to be tied to measurable and time-bound goals. Nutrition/health commitments made by companies fall into the following 4 broad categories: *1*) establishing nutrition profiles (e.g., ensuring a certain percentage of product portfolio meets company-specific or third-party nutrient profiles), *2*) food safety (typically centered on adherence to a third-party safety protocol, such as Global Food Safety Initiative standards [56]), *3*) labeling (transparency on nutrition facts panels, portion guidance and front-of-pack information, etc.), and *4*) ingredients (e.g., reducing certain ingredients from product portfolios, such as sodium, artificial colors and/or flavors).

Overall, the number and distribution of sustainability targets within each food systems domain varied across third-party frameworks, countries, or companies. This landscape assessment generally demonstrates a disproportionate representation of environmental commitments/goals and metrics compared with those for nutrition/health, most prominently evident when assessing sustainability targets of countries and companies. Importantly, the nature of this audit is limited to reflect only the number of commitments made by these entities, but not the quality, scope, or rationale of the commitments and associated metrics and indicators. Future work is needed to evaluate the reach and scale of each of these commitments and metrics, as well as to understand the context behind differences in sustainability targets across third-party frameworks, countries, or companies. Nevertheless, establishing more balanced commitments across the 4 domains of sustainability would help ensure that diet-sustainability models more fully capture the trade-offs of dietary patterns to sustainability targets [22]. This landscape approach is therefore a useful resource to support dialog and identification of opportunities for third-party frameworks, countries, and companies to more uniformly and effectively target sustainability progress across all 4 domains of food system sustainability.

## “Modeling” the Road Ahead: Gaps and Opportunities in Developing Robust Sustainability Measurement Tools for Evidence-Based Decision Making

**FIGURE 2:**
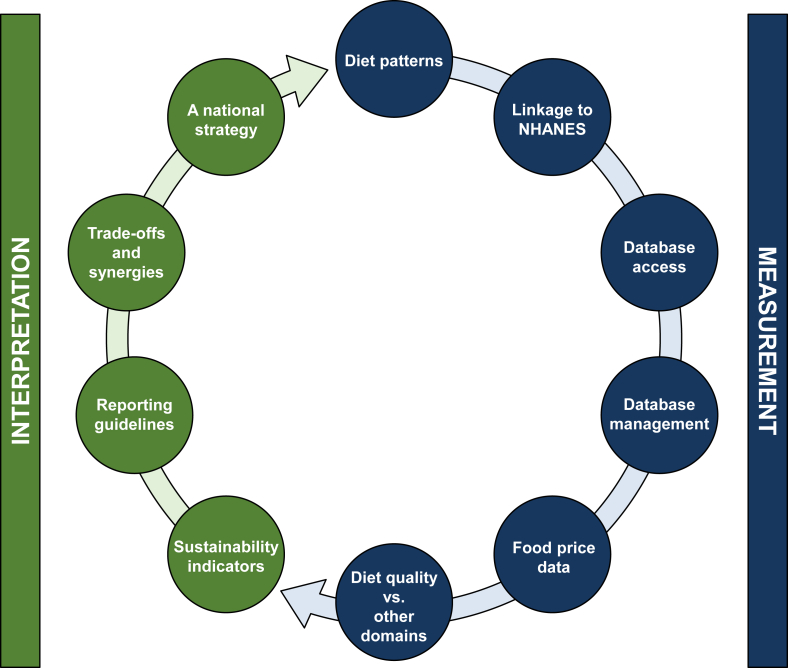
Summary of 10 priorities for supporting robust measurement tools and accurate interpretation of diet-sustainability analyses.

Discussed below are 10 priorities for addressing gaps and opportunities to advance diet-sustainability modeling research to support evidence-based guidance on sustainable diets (Figure 2). These priorities are focused on measurement of diet sustainability and interpretation of the evidence, and are focused on the United States food system, although many of these priorities apply to international food systems as well.

### Six priorities for supporting robust measurement tools for diet-sustainability analyses

#### Diet patterns

Diet-sustainability analyses evaluate the association of sustainability outcomes with either theoretical or actual dietary patterns. Modeling the relationship of theoretical dietary patterns with sustainability indicators, such as estimating the change in land use if all Americans adhered to the Dietary Guidelines for Americans [5], can be useful to explore and test the myriad of scenarios that are possible in our complex food systems. However, there are also many advantages to conducting analyses with databases of actual consumption patterns [57], a prominent example being What We Eat In America, the dietary intake component of the NHANES [58]. In particular, utilizing data from a sample population generates a wide distribution of food intake patterns that can be used to measure interindividual variability in food intake (and thus sustainability outcomes), allowing for statistical testing of differences between diet patterns and applicability to actual consumer food choices.

#### Linkage to NHANES

There are several high-quality sources of actual dietary data from sample populations, such as the Nurses’ Health Study [59] and Health Professionals Follow-Up Study that have been linked to environmental outcomes [60]. Nevertheless, nationally representative dietary data (i.e., NHANES) is a vital resource that should be leveraged for diet-sustainability research, and could also significantly advance scientific progress with linkage to sustainability indicators. Foundational to the development of federal policy [61], the maintenance of the NHANES database is under the purview of the federal government and coordinated efforts to link these dietary data to data on environmental, economic, and social outcomes would ensure access to high-quality, timely, and publicly available data for research purposes.

Current examples of nonfederal databases that link to NHANES data include dataFIELD [62,63], although this resource is currently limited in its representation of United States food systems and further efforts are needed to incorporate sustainability data from the postagricultural stage of the food system. Emphasis on development and maintenance of databases that link food system sustainability indices across the 4 domains to NHANES data will be instrumental for United States policymakers to develop relevant and feasible recommendations for Americans.

#### Database access

Although publicly available data are undeniably beneficial to spur scientific progress and enhance transparency of research in a given field, it is important to call attention to the realistic challenges involved. Of note, early career scientists frequently rely on exclusive use of their data to meet rigorous publication and tenure benchmarks. It follows that revolutionizing incentives beyond professional and social pressure to make data publicly available will likely be needed to realize widespread adoption of this research priority. Furthermore, funding support is needed for foundational but often overlooked research initiatives, such as database curation and management/updates.

#### Database management

An interrelated priority to database access, a fundamental input for diet-sustainability modeling, is access to not only high-quality but also current data. The Food Commodity Intake Database [64] and the Food Intakes Converted to Retail Commodities Database [65] are 2 leading food composition databases maintained by the federal government that are available for use by sustainability modelers; yet, these data have not been updated since 2010 and 2008, respectively. Updates to the Food Commodity Intake Database have been performed by academic scientists [66]; however, identifying and employing strategies to maintain these databases and improve the transparency of how these databases are developed could help overcome this significant barrier to ongoing sustainability modeling.

#### Food price data

Another gap related to database needs for modeling across the 4 domains of sustainability is the lack of food price data. Food spending outside of the home (known as Food Away From Home) notably accounts for >50% of Americans' food spending at the population level [67], yet high-quality individual-level data are not available. Furthermore, although independent scientists have previously estimated individual-level Food Away From Home to link with NHANES based on data derived from National Household Food Acquisition and Purchase Survey [68,69], this database is outdated as of 2013 [69]. The National Strategy on Hunger, Nutrition, and Health includes plans to release a new iteration of National Household Food Acquisition and Purchase Survey [70].

#### Diet-sustainability hypothesis

This hypothesis posits that there is a positive relationship between diet quality and sustainability outcomes. Data suggest that in some cases improved diet quality can reduce GHGe [57]. However, less is known about the impact of diet quality on the myriad of other indicators of environmental health (biodiversity, soil erosion, nutrient cycling, etc.), and some research suggests that there may be trade-offs between environmental indicators [71]. Further research is also needed to better understand how results are impacted when assessing different measures of diet quality (e.g., Healthy Eating Index, Alternate Healthy Eating Index, or Nutrient Rich Foods Index) [[71], [72], [73]]. As an example, Conrad et al. [71] found that higher scores on the Healthy Eating Index-2015 were associated with no change in the use of fertilizer nutrients but increased use of pesticides and irrigation water, whereas higher scores on the Alternate Healthy Eating Index-2010 were associated with lower use of fertilizer nutrients but no change in the use of pesticides and irrigation water. In a separate study, Conrad et al. [57] found that more closely meeting the dietary recommendations of the Dietary Guidelines for Americans (i.e., higher Healthy Eating Index-2015 scores) reduced diet-related GHGe but raised costs. Understanding these relationships has important implications for public health messaging efforts.

### Four priorities for supporting accurate interpretation of diet-sustainability analyses

#### Sustainability indices

Few studies evaluate >2 sustainability domains [74,75]. Given the need to measure the impact of food production and dietary patterns on all 4 domains of sustainability—as well as measure the impact on a multitude of indicators within each domain—further research efforts may focus on developing indices that represent multiple outcomes. These metrics are similar in concept to diet quality indices that capture the multidimensional nature of dietary patterns, which include many foods, food groups, and nutrients. Great care and rigor will be needed to identify which domains and indicators to include (and exclude), and how to score each of them [75]. This field is in infancy; as efforts progress to develop diet-sustainability measurement tools and tactics, scientific dialog will be needed to fully evaluate the effectiveness of composite metrics/indices inclusive of all 4 food systems domains to aid evidence-based decision making [74,75].

#### Reporting guidelines

A viable strategy to promote international alignment on best practices for conducting and reporting sustainability research is through the development of reporting guidelines. STROBE Nutrition Guidelines provide an example framework for nutrition research that could support such an initiative in the sustainability field [76]. Because of the complex nature of sustainability modeling, reporting guidelines could provide much needed transparency for several methodologic aspects, including but not limited to management of inflated error estimates that occurs when merging multiple databases; assumptions made in computational modeling; details of how models are being validated and sensitivity analyses conducted; and a standard for the number and quality of sustainability domains and indicators to be included in the analysis for the modeling to be defined as “sustainability research.”

#### Trade-offs and synergies

Although this field of study is still emerging, it is important to note that optimization across all domains and indicators of sustainability will be challenging, and perhaps not possible. For example, previous research already points to the probability that benefits across one domain may necessitate compromises in another [57]. Accordingly, policymakers and other agrifood decision makers across sectors will be expected to make difficult decisions when prioritizing certain trade-offs to ensure certain synergies in a context-dependent fashion and to align with societal values. These values may vary by context, including space and time. Moving forward, transparency in the dissemination and interpretation of results to clearly define synergies and trade-offs of diets can support evidence-based policy making in a manner that is respectful of diverse cultural and societal values.

#### A national strategy

The launch of the National Strategy on Hunger, Nutrition, and Health in 2022 is, at its core, a sustainability strategy that includes priorities to address all 4 domains of sustainable food systems [70]. This strategy therefore can be viewed as a blueprint for stakeholders across sectors to bridge existing gaps in sustainability with maximum impact by working toward this common set of priorities.

Another topical issue central to sustainability policy is the dialog surrounding the appropriateness of sustainability recommendations within the United States Dietary Guidelines for Americans [77]. Irrespective of whether or not sustainability guidance should fall within the purview of the Dietary Guidelines for Americans, the 9 priorities discussed above underscore the complexity of this emergent field of research and the limited science available that impedes accurate and holistic recommendations. Before sustainability can be considered as an element of nutrition guidance, it is thus paramount to first establish a clear relationship between diet quality and sustainability outcomes.

To summarize, there are several challenges, unknowns, and opportunities to develop robust sustainability measurement tools that accurately capture the relationship between dietary patterns and the multifaceted dimensions of sustainable food systems. Beyond the scope of this discussion, other reforms to benefit academic-based scientific research could target the expectations of publishing research, training, and education of the early career workforce as well as providing opportunities for faculty positions and funding. Even so, there are many more priorities that deserve attention and that need to be addressed to fully understand the diet-sustainability nexus and ultimately achieve planetary health.

## Conclusion

It is clear that current food systems are facing challenges and that global collective action is urgently needed to meet acute and long-term needs for public nutrition and health, environmental stewardship, economic sustainability, and social needs. It is thus no surprise that calls for inclusion of sustainability guidelines in dietary guidance by stakeholders across sectors have increasingly gained attention by policymakers; indeed, adoption of sustainability considerations has already begun to appear in certain national food-based dietary guidelines around the world [78].

At the same time, this article provides perspective on the significant gaps that currently exist in the emerging and complex field of diet-sustainability modeling that plays a foundational role in evidence-based decision making to achieve food systems transformation. Although significant steps have been made to measure and evaluate how our food system impacts a limited suite of metrics for environmental sustainability, assessment of the complementary roles of nutrition/health, economic, and/or social considerations within this context is critically lacking. Because global efforts continue to seek impactful strategies to realize a transformation of our food system, it will be critical to align on robust measurement tools to holistically assess the contributions of food production and dietary patterns to sustainable diets. Furthermore, special attention to communicate to the public the trade-offs and synergies of diets on measures of sustainability will also require careful education efforts, message crafting and testing and feedback by the agrifood community and the public to minimize unintended consequences for public health.

## Author contributions

The authors’ responsibilities were as follows – KB, ALU: conceptualized the content of the symposium; ALU: drafted the manuscript based on the symposium content; and all authors: developed the content of this manuscript, participated in the editing process, confirmed the final content, and read and approved the final manuscript.

## Funding

The authors reported no funding received for this study. The ASN Nutrition 2023 session that this article is based on was supported by National Dairy Council. This support included an honorarium for ZC.

## Conflict of interest

ALU, MAP, and KB were employees of National Dairy Council at the time this article was written. The use of brands and/or images of branded products is intended only to provide examples of concepts being discussed and does not imply endorsement of any brand or product. ZC has research awards from the Jeffress Trust Awards Program in Research Advancing Health Equity and the USDA for projects unrelated to this article. The other author reports no conflicts of interest.
